# Improved Accuracy of Lymph Node Staging and Long-Term Survival Benefit in Colorectal Cancer With *Ex Vivo* Arterial Methylene Blue Infiltration

**DOI:** 10.3389/pore.2022.1610742

**Published:** 2022-10-18

**Authors:** Nóra Suszták, István Besznyák, Kálmán Almási, Attila Bursics, Dóra Kelemen, David W. Borowski, Balázs Bánky

**Affiliations:** ^1^ Faculty of Medicine, Semmelweis University, Budapest, Hungary; ^2^ Department of Surgery, St. Imre University Teaching Hospital, Budapest, Hungary; ^3^ Department of Surgery, Uzsoki Street Hospital, Budapest, Hungary; ^4^ Department of Pathology, Aladar Petz County Teaching Hospital, Győr, Hungary; ^5^ Department of Pathology, Uzsoki Street Hospital, Budapest, Hungary; ^6^ Department of Surgery, Welwitschia Hospital, Walvis Bay, Namibia; ^7^ Department of Surgery, Transplantation and Gastroenterology, Semmelweis University, Budapest, Hungary

**Keywords:** colorectal cancer, overall survival, methylene blue, nodal staging accuracy, lymph node staging

## Abstract

**Introduction:** *Ex vivo* methylene blue (MB) injection into the main supplying arteries of the colorectal specimen after surgical removal is an uncomplicated technique to support lymph node harvest during pathological evaluation. The primary aim of this randomized, interventional, bicentric trial was to evaluate the impact of MB injection on lymph node yield, with secondary aims assessing the accuracy of lymph node staging and the effect on 5-year overall survival for patients undergoing resection of colorectal cancer.

**Methods:** In the study period between December 2013 and August 2015, 200 colorectal resections were performed at two independent onco-surgery centers of Hungary. Following surgical resection, each specimen was randomly assigned either to the control (standard pathological work-up) or to the MB staining group before formaldehyde fixation. Patient-level surgical and clinical data were retrieved from routinely collected clinical datasets. Survival status data were obtained from the National Health Insurance Fund of Hungary.

**Results:** A total of 162 specimens, 82 in the control and 80 in the MB groups, were included for analysis. Baseline characteristics were equally distributed among study groups, except for specimen length. Both the median of total number of lymph nodes retrieved (control 11 ± 8 [0–33] nodes vs. MB 14 ± 6 [0–42] nodes; *p* < 0.01), and the ratio of cases with at least 12 removed lymph nodes (36/82, 43.9% vs. 53/80, 66.3%; *p* < 0.01) were higher in the MB group. The rate of accurate lymph node staging was non-significantly improved. As for rectal cancer, nodal staging accuracy (16/31, 51.6% vs. 23/30, 76.7%; *p* = 0.04) and the proportion with minimum 12 lymph node retrieval (7/31, 22.6%, vs. 18/30, 60%; *p* < 0.01) was improved by MB injection. In Mantel–Cox regression, a statistically significant survival benefit with methylene blue injection at 5 years post-surgery was proven (51.2% vs. 68.8%; *p* = 0.04).

**Conclusion:** In our experience, postoperative *ex vivo* arterial methylene blue injection appears to be an uncomplicated technique, improving lymph node yield and decreasing the chance of insufficient nodal staging. The technique might also associate with a 5-year overall survival benefit.

## Introduction

Colorectal cancer (CRC) is the second most common cause of cancer death worldwide [1]. Currently, the cornerstone of treatment is surgery with curative intent, and as such, *en bloc* removal of the local lymph node (LN) field is a key aspect of the operation [2]. Operative perfection of lymph node clearance, called complete mesocolic excision (CME) in the case of colon lesions, and total mesorectal excision (TME) in the case of rectal tumors, is associated with survival benefit and local recurrence reduction [3, 4]. Accurate lymph node staging based on histopathologic examination of each available lymph node (or as many nodes as possible) is recommended both as a prognostic tool and decision basis of adjuvant chemotherapy [2, 5, 6]. In 2009, the *Union for International Cancer Control* (UICC) recommended at least 12 lymph nodes to be harvested and reported, in order to adequately exclude node-positive disease [7]. Differentiating between stage II and stage III colorectal cancer is paramount, as most stage II CRC requires no adjuvant treatment, while surgical removal of stage III cancers with at least one metastatic lymph node or presence of extranodal tumor deposits should usually be followed by chemotherapy [2]. In the case of insufficient lymph node retrieval from the surgical specimen, node-negative tumors leave some uncertainty regarding the reliability of a pN0 stage. Incorrect classification may lead to inadequate postoperative management either way, which might contribute to a worse prognosis [8]. The estimated understaging rates may be as high as 6% in some studies [9, 10]. In particular, the subset of patients who undergo pre-operative chemo-irradiation may have fewer lymph nodes due to the effect of neoadjuvant treatment (NAT), but may also have better outcomes despite suboptimal lymph node harvest and examination [11]. Other studies have previously demonstrated that an increased number of histopathologically examined lymph nodes seems to be associated with better long-term survival [8, 12, 13].

The obvious need for accurate lymph node staging has led to the development of several techniques to improve the lymph node yield. The traditional visualization and manual palpation of the mesocolon for lymph nodes is performed first by the pathologist. However, 80% of mesorectal lymph nodes are smaller than 3 mm, and at least half of metastatic lymph nodes are smaller than 5 mm [14, 15]. Additional methods employed by the pathologist include fat clearance techniques with xylene, alcohol or acetone, which can improve visualization of lymph nodes within the mesocolic or mesorectal fat. Although these methods have been demonstrated to increase lymph node harvest, they can be time-consuming, expensive and harmful for personnel due to toxic components [16, 17]. In some countries, pathologist assistants are employed to enhance the number of dissected lymph nodes, but due to increased costs, this strategy may be unaffordable in some healthcare systems [18].

Methylene blue (MB) injection into the main supplying artery of the removed colorectal specimen is a low-cost, simple and non-hazardous maneuver [19–21]. It results in accumulation of the blue dye in the vessels, lymphatic channels and lymph nodes, providing the pathologist with a good color contrast between nodes and surrounding fat tissue. This *ex vivo* method was first introduced by Märkl et al. in 2007, and multiple groups have adopted this approach considering its feasibility and affordability [9, 20–33]. Our study aimed to examine the method of MB injection in a randomized setting within an Eastern European healthcare system. The primary outcome of the study included the overall LN harvest, and the proportion of accurately staged patients. The secondary aim was to analyze the effect of lymph node staging on 5-year overall survival of recruited patients.

## Materials and Methods

### Study Sites

A randomized, interventional trial was conducted at two independent onco-surgery centers (St. Borbala Hospital in Tatabánya and Uzsoki Street Hospital in Budapest, Hungary). Both high-volume colorectal centers ran more than 150 elective colorectal surgical cases *per annum* during the study period of 20 months between December 2013 and August 2015. Both sites shared the same enrollment, randomization and specimen processing protocols.

### Power Calculation

An *a priori* power calculation was performed for primary outcome measures (total lymph node count and nodal staging accuracy), based on published data and our unpublished local pilot [20]. At least 85 cases on each arm were required to detect a 20% improvement of lymph node yield between study arms, with a 5% level of statistical significance and 80% statistical power. We calculated with a 10% case dropout, therefore we planned 200 cases to recruit with a 1:1 randomization ratio at both sites.

### Randomisation of Specimens

At each site, colorectal cancer cases with prior histopathological verification from colonoscopic biopsy specimens, or without prior histopathological verification but with high clinical suspicion by colonoscopic presentation and computed tomographic imaging, undergoing segmental colon or rectum resection were enrolled in the study. Surgically removed specimens were randomly allocated to either MB or conventional specimen processing as controls in the operating room promptly after resection, by choosing one of 100 pre-prepared, sealed envelopes at each site.

### Specimen Processing

**FIGURE 1:**
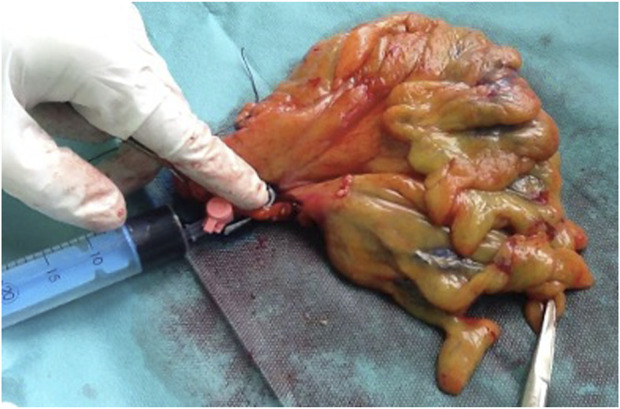
*Ex vivo* methylene blue solution injection into the supplying artery of the colorectal specimen after surgical removal.

Native, freshly removed colorectal specimens from the control group were directly placed in routine 10% buffered neutral formaldehyde solution for 48–72 h. Specimens of the MB group were laid out, and the main supplying artery or arteries were cannulated with a 20 G cannula, according to the standard central vascular ligation technique. Then, 50 mg of MB was diluted with 30 ml saline, and the solution was injected into the main arterial trunk(s) until the dye appeared on the cut surfaces of the specimen (Figure 1). The dyeing process was executed in the operating room on a back table. Finally, specimens were placed in 10% buffered neutral formaldehyde solution for 48–72 h. All specimens were then processed according to pathological routine following standard practice.

### Pathological Evaluation

After visual inspection and palpation, pathologists harvested as many lymph nodes from each specimen as possible. MB staining, the number of extracted lymph nodes, and the number of metastasis-positive lymph nodes were documented in the pathological report. Accurate lymph node staging was defined as any case with at least one positive lymph node, report of extranodal tumor deposit, or pN0 stage based on at least 12 examined lymph nodes. As a quality indicator of work-up, a ratio of cases with at least 12 lymph nodes retrieved was also evaluated retrospectively.

### Data Processing

A separate, anonymized database was built for data collection. Demographic data, tumor localization, surgical access, concomitant neoadjuvant therapy, operating surgeons, evaluating pathologists, details of the pathological report (TNM stage, histology grade, CME/TME surgical grade, specimen length, total number of dissected lymph nodes and metastatic lymph nodes) were collected. Both the results of randomization and pathological results were collected from the hospital documentation systems. Additional pre-planned appointments of patients, besides the routine surgical and oncological follow-ups were not arranged. Further follow-up data of included patients were extracted from the hospital documentation systems, and after 5 years of study completion, the survival status and time to death within 60 months post-surgery were collected electronically from the database of the National Health Insurance Fund of Hungary.

### Statistical Analysis

Continuous variables with normal distribution were analyzed with their mean ± standard deviation, while skewed distributed variables were reported as median with interquartile range (IQR) and minimum-maximum ranges. Categorical variables were reported as absolute numbers (n) with relative percentages (%) of the given group. Normal distribution of continuous variables was determined by the Shapiro–Wilk test. Continuous variables were compared using Student’s *t*-test or the Mann–Whitney U-test. Categorical variables were compared using chi-squared tests. For correlation analysis, Pearson and Spearman Rho tests were used. The effect of intervention on survival was studied using a Mantel–Cox regression analysis, based on Kaplan–Meier curves and log-rank test. Level of statistical significance was set at a two-tailed *p* value of <0.05. All statistical analyses were done using SPSS version 24.0 (SPSS Inc., Chicago, IL, United States).

### Primary and Secondary Outcomes

Primary endpoints of the study included 1) the total number of harvested lymph nodes, and 2) the rate of accurate lymph node staging. Secondary outcome was overall survival at 5 years post-operatively for each group.

## Results

### Patient Enrollment

**FIGURE 2:**
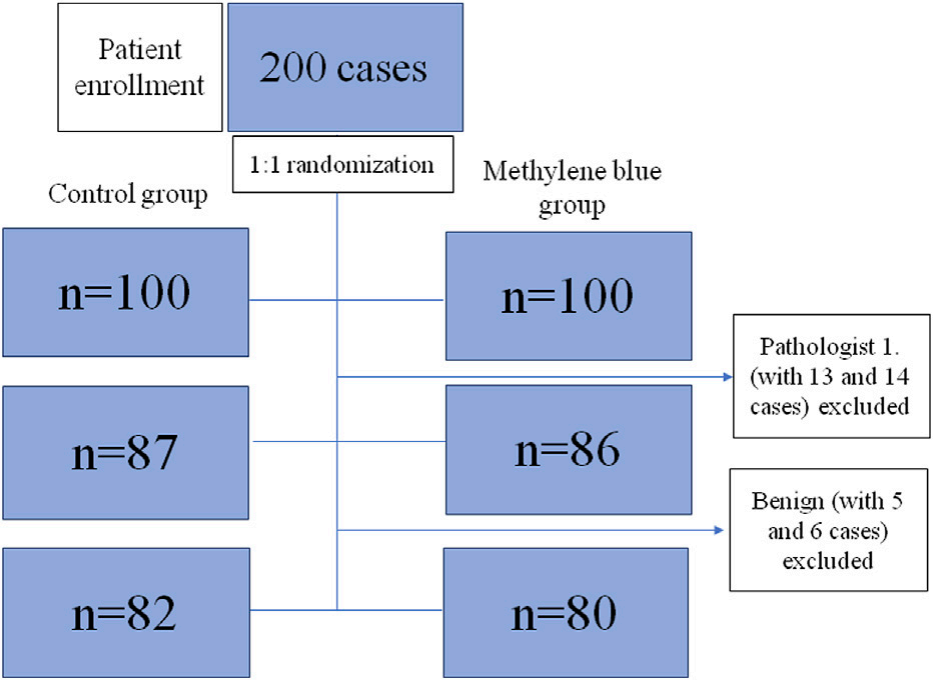
Flow diagram of study enrollment.

The Patient enrollment flow diagram is reported in Figure 2. Over the period of 20 months, one hundred cases of elective colon or rectal resections were included at each of the two hospitals. One of the pathologists decided not to participate in the study, thus his cases were excluded (13 cases and 14 cases in the control and MB groups, respectively.) Likewise, a further 11 cases with benign histopathology were also excluded, five from the control and six from the MB group. A total of 162 specimens, 82 in the control and 80 in the MB groups, were included.

### Baseline Demographic and Clinical Characteristics of the Two Groups

MB and control groups were found to be statistically comparable regarding patient age, sex, tumor site, type of operation, laparoscopic or open access surgery, TNM staging, CME/TME surgical grade, variability of operating surgeons and pathologists, as well as hospital site (Table 1). Although the median specimen length was significantly higher in the MB group (25 ± 10 cm vs. 22 ± 11 cm; *p* = 0.03), specimen length and total number of retrieved lymph nodes were proved to be independent in Pearson’s correlation test (*p* = 0.58) and Spearman’s Rho correlation test (*p* = 0.55).

**TABLE 1:** Distribution of baseline clinical and oncological parameters among included patients, grouped by study intervention.

Parameter	Control group (*n* = 82)	MB group (*n* = 80)	*p* value
Age (years, median ± IQR, min–max)	73 ± 15 (32–88)	73 ± 16 (40–92)	0.81^a^
Sex (n, %)			
Female	40 (48.8%)	40 (50%)	0.88^b^
Male	42 (51.2%)	40 (50%)	
Tumor site (n, %)			
Cecum	11 (13.4%)	9 (11.3%)	0.91^b^
Ascending colon	11 (13.4%)	7 (8.8%)	
Hepatic flexure	3 (3.7%)	3 (3.8%)	
Transverse colon	3 (3.7%)	5 (6.3%)	
Splenic flexure	1 (1.2%)	1 (1.3%)	
Descending colon	0 (0%)	1 (1.3%)	
Sigmoid colon	22 (26.8%)	24 (30.0%)	
Rectum	31 (37.8%)	30 (37.5%)	
Operation type (n, %)			
Right colectomy	26 (31.7%)	20 (25%)	0.23^b^
Extended right colectomy	0 (0%)	4 (5%)	
Transverse colectomy	2 (2.4%)	0 (0%)	
Left colectomy	2 (2.4%)	1 (1.3%)	
Sigmoid colectomy	2 (2.4%)	3 (3.8%)	
Anterior rectum resection	44 (53.7%)	43 (53.8%)	
Abdomino-perineal excision of the rectum	6 (7.3%)	9 (11.3%)	
Surgical access (n, %)			
Open	36 (43.9%)	37 (46.3%)	0.11^b^
Laparoscopic	45 (54.9%)	37 (46.3%)	
Converted	1 (1.2%)	6 (7.5%)	
T stage (n, %)			
Tis	1 (1.2%)	1 (1.3%)	0.48^b^
T1	6 (7.3%)	5 (6.3%)	
T2	10 (12.2%)	10 (12.5%)	
T3	57 (69.5%)	61 (76.3%)	
T4	8 (9.8%)	3 (3.8%)	
N stage (n, %)			
N0	47 (57.3%)	43 (53.8%)	0.89^b^
N1	24 (29.3%)	26 (32.5%)	
N2	11 (13.4%)	11 (13.8%)	
M stage (n, %)			
M0	71 (86.6%)	76 (95%)	0.06^b^
M1	11 (13.4%)	4 (5%)	
AJCC Stage			
0	1 (1.2%)	1 (1.3%)	0.37^b^
I	11 (13.4%)	15 (18.8%)	
II	30 (36.6%)	27 (33.8%)	
III	29 (35.4%)	33 (41.3%)	
IV	11 (13.4%)	4 (5%)	
Pathologists participating in the study (n, %)			
P1	15 (18.3%)	13 (16.3%)	0.98^b^
P2	5 (6.1%)	5 (6.3%)	
P3	18 (22%)	20 (25%)	
P4	4 (4.9%)	7 (8.8%)	
P5	3 (3.6%)	3 (3.8%)	
P6	5 (6.1%)	4 (5%)	
P7	3 (3.6%)	3 (3.8%)	
P8	9 (11%)	9 (11.3%)	
P9	7 (8.5%)	4 (5%)	
P10	13 (15.8%)	11 (13.8%)	
P11	0 (0%)	1 (1.3%)	
Surgeons participating in the study (n, %)			
S1	4 (4.9%)	6 (7.5%)	0.46^b^
S2	17 (20.7%)	17 (21.3%)	
S3	11(13.4%)	4 (5%)	
S4	4 (4.9%)	7 (8.7%)	
S5	0 (0%)	2 (2.5%)	
S6	2 (2.4%)	3 (3.8%)	
S7	2 (2.4%)	3 (3.8%)	
S8	0 (0%)	1 (1.3%)	
S9	13 (15.8%)	10 (12.5%)	
S10	1 (1.2%)	1 (1.3%)	
S11	1 (1.2%)	0 (0%)	
S12	1 (1.2%)	0 (0%)	
S13	2 (2.4%)	4 (5%)	
S14	5 (6.1%)	6 (7.5%)	
S15	1 (1.2%)	4 (5%)	
S16	1 (1.2%)	0 (0%)	
S17	0 (0%)	1 (1.3%)	
S18	5 (6.1%)	5 (6.3%)	
S19	3 (3.6%)	1 (1.3%)	
S20	7 (8.5%)	2 (2.5%)	
S21	0 (0%)	2 (2.5%)	
S22	2 (2.4%)	1 (1.3%)	
Included patients at each site (n, %)			
H1	35 (42.7%)	35 (43.8%)	0.87^b^
H2	47 (57.3%)	45 (56.3%)	
Neoadjuvant therapy received (n, %)	16 (19.5%)	14 (17.5%)	0.74^b^
Specimen length (centimeters, median ± IQR, min–max)	22 ± 11 (5–52)	25 ± 10 (9–83)	**0.03** ^a^

The bold values mean that those vales has reached a level of significancy.

H1: St. Borbala Hospital, Tatabánya, H2: Uzsoki Street Hospital, Budapest.

^a^ Mann-Whitney U test.

^b^ Chi-square test.

### Lymph Node Count and Nodal Staging Accuracy

Parameters of lymph node count and nodal staging accuracy are described in Table 2. The total number of lymph nodes retrieved from specimens was higher in the MB group, compared to the control group. Separate analysis of colon and rectal cancer subgroups showed that improvement of the lymph node yield is mainly focused to the rectal subgroup, while in the colon subgroup, this improvement did not reach statistical significance. The ratio of cases with at least 12 retrieved lymph nodes was significantly higher in the MB group compared to the control group. There was a tendency for a higher rate of accurate lymph node staging, as an improvement was noted in the MB group (81.3%), compared to the control group (69.5%). Lymph node staging accuracy was significantly higher among rectal cases in the MB group, while no statistical difference was found among colonic cases between groups. During analysis, 30 cases of rectal cancers were operated on after neoadjuvant chemo-irradiation. Although the mean of the positive lymph node count was unchanged by the intervention, nodal staging accuracy showed a statistically non-significant tendency for improvement, while the total lymph node counts and the proportion of ≥12 lymph node retrieval reached statistically significant improvement by MB injection.

**TABLE 2:** Lymph node count and nodal staging accuracy, grouped by experimental arms and tumor site.

Lymph node counts	Control group (*n* = 82)	MB group (*n* = 80)	*p* value
Total lymph node count (nodes, median ± IQR, min–max)			
Total (rectum + colon)	11 ± 8 (0–33)	14 ± 6 (0–42)	**<0.01** ^a^
Colon	12 ± 7 (4–33)	14 ± 8 (2–42)	0.13^a^
Rectum (total)	8 ± 8 (0–23)	12.5 ± 8 (2–28)	**0.01** ^a^
Rectum (neoadjuvant)	6.5 ± 6 (0–23)	12 ± 9 (2–19)	**0.04** ^a^
Rectum (without neoadjuvant)	10 ± 11(2–20)	13.5 ± 6 (6–28)	0.15^a^
Positive lymph node count (nodes, mean ± SD, min-max)			
Total (rectum + colon)	1.6 ± 3.6 (0–20)	1.5 ± 2.4 (0–12)	0.35^a^
Colon	2 ± 4.4 (0–20)	1.5 ± 2.6 (0–12)	0.69^a^
Rectum (total)	0.8 ± 1.64 (0–6)	1.3 ± 2.1 (0–9)	0.31^a^
Rectum (neoadjuvant)	0.8 ± 1.6 (0–6)	1 ± 2.4 (0–9)	0.98^a^
Rectum (without neoadjuvant)	0.9 ± 1 (0–5)	1.6 ± 3 (0–5)	0.28^a^
Proportion with ≥12 nodes examined, per total number of lymph nodes (n, %)			
Total (rectum + colon)	36/82 (43.9%)	53/80 (66.3%)	**<0.01** ^b^
Colon	29/51 (56.9%)	35/50 (70%)	0.17^b^
Rectum (total)	7/31 (22.6%)	18/30 (60%)	**<0.01** ^b^
Rectum (neoadjuvant)	2/16 (12.5%)	8/14 (57.1%)	**0.01** ^b^
Rectum (without neoadjuvant)	5/15 (33.3%)	10/16 (62.5%)	0.10^b^
Accurate nodal staging, per total (n, %)			
Total (rectum + colon)	57/82 (69.5%)	65/80 (81.3%)	0.08^b^
Colon	41/51 (80.4%)	42/50 (84%)	0.64^b^
Rectum (total)	16/31 (51.6%)	23/30 (76.7%)	**0.04** ^b^
Rectum (neoadjuvant)	7/16 (43.8%)	9/14 (64.3%)	0.26^b^
Rectum (without neoadjuvant)	9/15 (60%)	14/16 (87.5%)	0.08^b^

The bold values mean that those values have reached a level of significancy.

^a^ Mann-Whitney U test.

^b^ Chi-square test.

### Survival Analysis

**FIGURE 3:**
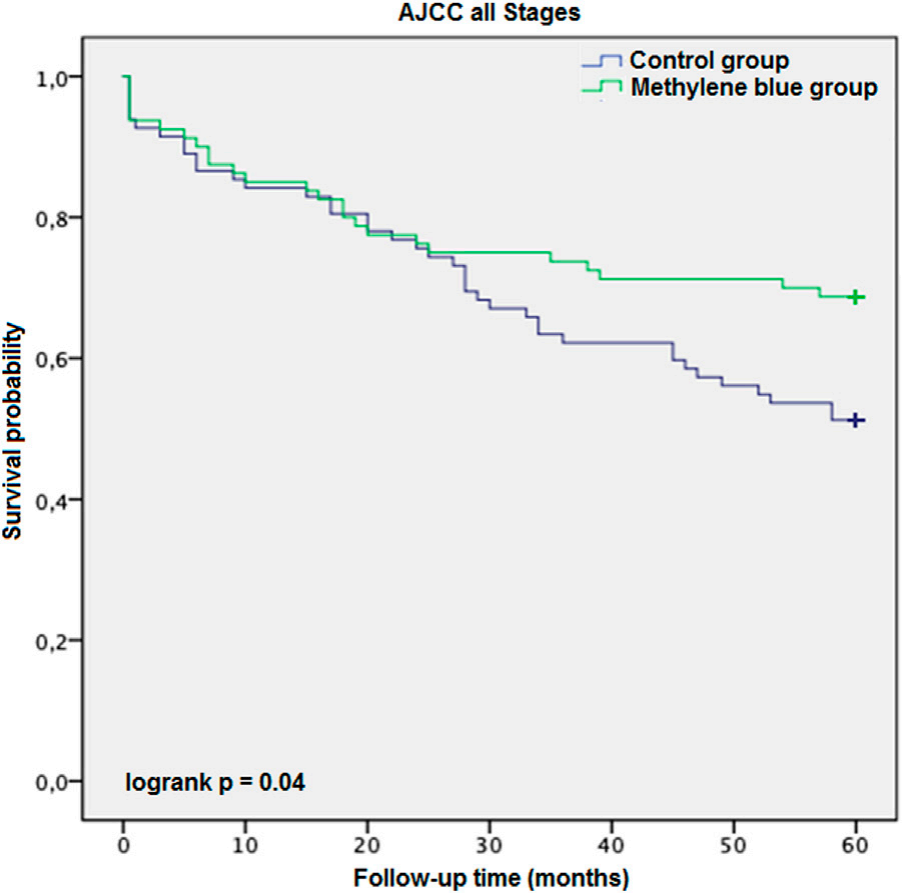
AJCC all stages description: Kaplan-Meier survival curves of all enrolled patients during the follow-up period, grouped by study intervention.

**FIGURE 4:**
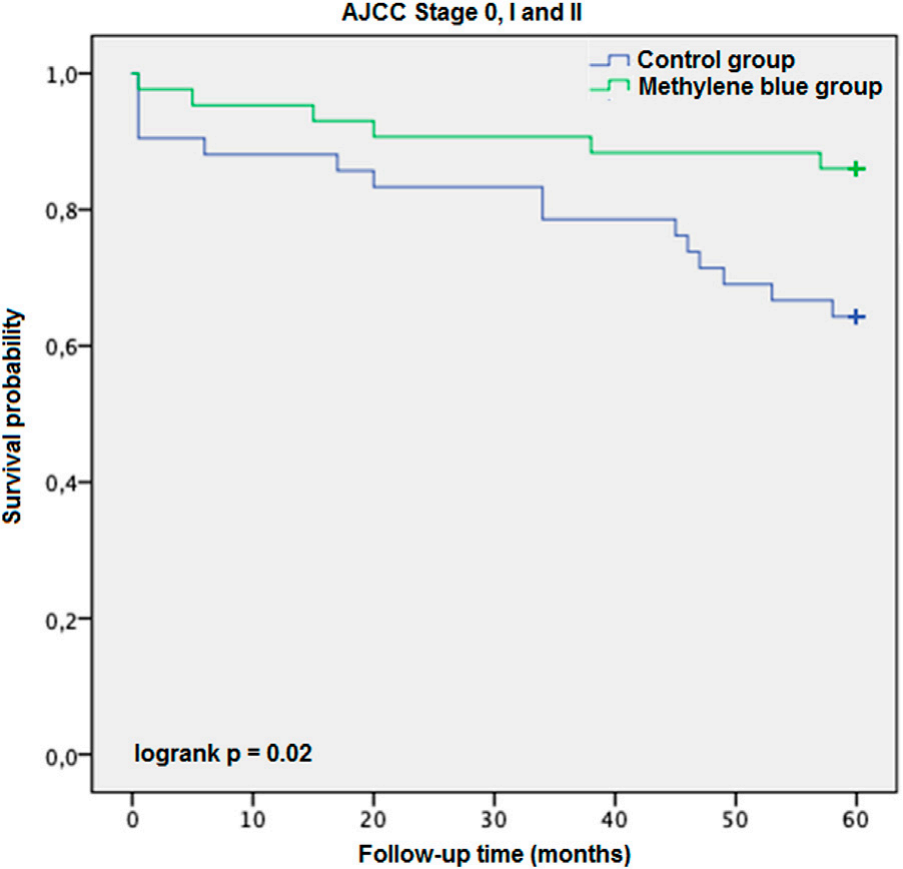
AJCC Stage 0, I and II description: Kaplan-Meier survival curves of enrolled patients with early stage colorectal cancer (AJCC Stages 0, I and II) during the follow-up period, grouped by study intervention.

**FIGURE 5:**
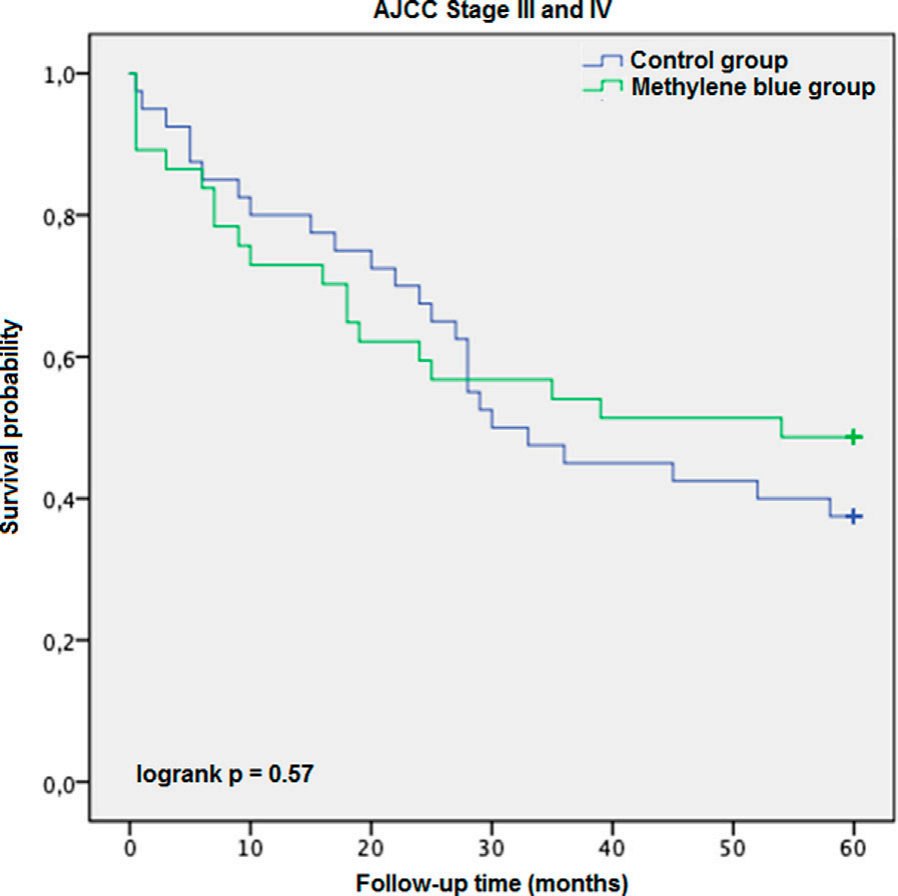
AJCC Stage III and IV description: Kaplan-Meier survival curves of enrolled patients with advanced colorectal cancer (AJCC Stages III and IV) during the follow-up period, grouped by study intervention.

Each case was censored at 60 months of follow-up. Non-stratified Kaplan-Meier curves of overall survival were undivided until ∼24 months, and clearly split afterwards (Figure 3). In Mantel-Cox regression, methylene blue injection showed a statistically significant survival benefit at 5 years after operation (51.2% vs. 68.8%; *p* = 0.04). In the stratified analysis, patients from the control group of early CRC stages (AJCC Stages 0, I and II) had a significantly lower overall survival, in contrast to the MB subgroup (64.3% vs. 86.0%; *p* = 0.02), while in advanced CRC stages (AJCC Stages III and IV), no statistically significant survival benefit could be observed (37.5% vs. 48.6%; *p* = 0.57) (Figures 4, 5).

## Discussion

There has been a controversial discussion in the literature about the necessary number of dissected lymph nodes during colorectal cancer pathological evaluation. The cut-off threshold varied from 6 to 20 extracted lymph nodes in different studies [34]. Conventionally, the UICC, the *American Joint Committee on Cancer*, the *National Quality Forum*, *National Comprehensive Cancer Network* and the *College of American Pathologists* recommend a minimum number of 12 lymph nodes for accurate staging, as the quality measure of both surgical lymph node clearance and pathological workup [34]. On the other hand, a recently published high volume study proposed a minimal cut-off value above 21 lymph nodes [8]. Regardless of the recommendations, the majority of dissected colorectal specimens does not reach the minimum recommended lymph node count, especially in cases of rectal cancer after neoadjuvant chemo-irradiation [12, 35, 36].

Our results are largely consistent with those found in the literature. Application of this method might result in an increase of lymph node yield [9, 19–33]. Although a tendency for increment could be observed in each subgroup, a statistically significant improvement was documented among rectum cancer cases but not for colonic cases.

Furthermore, methylene blue staining provided a significant increase in the rate of sufficient (≥12) lymph node detection in our study, as a quality measure for pathological work-up. In the control group, lymph node harvest reached the minimal number in less than half of all cases, while with MB staining, two-thirds of the cases were sufficiently reported. This effect was again more pronounced in the rectal cancer subgroup. Reviewing the related articles from the literature, the rate of insufficient lymph node harvest could possibly be diminished, even close to zero, with the help of MB staining [9, 20–24, 27].

Lymph node staging accuracy is a complex term, covering the minimal number (≥12) of dissected lymph nodes, and N1c (with extranodal tumor deposit) stages and others with at least one positive lymph node (N1–2). In our experience, the rate of accurate lymph node staging by methylene blue injection was markedly improved, but this tendency did not reach the level of statistical significance. In further subgroup analysis, lymph node staging accuracy significantly improved among rectal cancer cases without neoadjuvant therapy, while no statistical difference was found in the colon cancer subgroup. Similar findings have been reported by other scientific groups [20, 21, 24, 27]. Regarding the small lymph nodes of the colorectal mesentery, there is great deal of inconsistency. Some authors, like Märkl et al. reported that most of the metastatic lymph nodes are simply noticeable by pathological examination [21]. Similarly, in the analysis of Reima et al., only 8% of the patients had metastasis in small-diameter (≤4 mm) lymph nodes, meaning that small lymph nodes may not be subject of major clinical interest [27]. On the other hand, other authors state that the majority (or at least half) of metastatic lymph nodes are located in lymph nodes smaller than 5 mm [37–39]. In these cases, methylene blue staining might help in the detection of these otherwise difficult-to-retrieve small metastatic lymph nodes.

Recently, a few trials have reported a significant increment of the metastatic lymph node count during the methylene blue technique, compared to the conventional lymph node dissection, leading to stage migration or upstaging [9, 30, 31]. For example, Jepsen et al. observed a significant increase in the rate of positive lymph nodes, resulting in an upstaging from UICC stage I or II to stage III, however in advanced cancer (T3/4), no upstaging was detected [31]. In addition, Liu et al. published a significantly raised rate of node positive lymph nodes resulting in an upstaging in 4 cases out of 66 [9]. In our study, in spite of the improved number of examined lymph nodes, this was not observable. Our experimental setting however was not designed to point out nodal stage migration.

It is well established that neoadjuvant chemo-irradiation negatively affects lymph node yield in rectal cancer cases, as preoperative treatment leads to fibrosis and lymph node size decrease in the affected area, causing a lack of properly dissectible lymph nodes [9,40–42]. However, this phenomenon may not negatively influence oncological outcomes, if we consider the decreased number and size of mesorectal lymph nodes as a favorable marker of neoadjuvant treatment effectivity [29]. This might be in line with the observation reported by Gurawaila et al., as fewer retrieved mesorectal lymph nodes correlated with effectiveness of oncotherapy after NAT [11].

Methylene blue injection has been successfully applied in the pathological investigation of rectal cancers in a few recent studies [20, 24, 29, 32]. Borowski et al., Klepšytė et al. and Münster et al. found that the use of MB injection led to a higher lymph node yield among patients who have received NAT, and the number of cases with insufficient lymph node detection was reduced, while no increase of the lymph node recovery in the subgroup of rectal cancer without preoperative radio-chemotherapy was observed [20, 29, 32]. Similar results were obtained in our research. In our experience, the use of MB injection showed a clinically relevant impact on the total lymph node count, the rate of sufficient (≥12) lymph node extraction, and lymph node staging accuracy in patient subgroups with rectal cancer.

Lymph node yield has a significant association with long term survival [43]. This is probably due to accurate nodal staging and the appropriate indication of adjuvant chemotherapy. Even one positive lymph node can cause a staging shift from stage II to stage III CRC, leading to different postoperative treatment [44, 45]. While stage II CRCs do not always require adjuvant treatment, stage III cancers are followed by adjuvant chemotherapy, as long as the patient is fit enough to complete it [44, 45]. Therefore, an insufficient lymph node extraction might contribute to a worse prognosis [8]. Although several methods have been introduced to increase the number of the dissected lymph node yield, most of them lack information on direct survival benefit [16–22, 24, 26, 27, 29–32]. We found that MB injection might be associated with improved 5-year overall survival among patients with colorectal cancer, undergoing colorectal resection. Similarly, Liu et al. have also investigated the overall survival after methylene blue injection in rectal cancer. However, their study showed no long-term benefit [9]. We observed no difference between the control and the MB arms until 24 months. After a period of a 2-year follow-up, Kaplan-Meier survival curves split up, and at 5 years postoperatively, the difference reached the level of statistical significance. Furthermore, in our stratified subgroup analysis, data suggested that cases with early CRC could profit the most from the MB staining method. The difference between the findings of Liu et al. and our group might be explainable by the alternation of adjuvant oncotherapy strategies among insufficiently staged cases. While Liu et al. reported that their colorectal multidisciplinary team rostered these patients for adjuvant chemotherapy, in our practice, we prefer not to treat patients with less than 12 negative lymph nodes. The necessity of adjuvant chemotherapy in stage II with insufficient (≤12) lymph node removal has a controversial interpretation in the literature. Current colorectal cancer guidelines recommend that early CRCs with less than 12 examined lymph nodes should be considered high-risk for recurrence, and are associated with increased mortality, and therefore, adjuvant chemotherapy should be administered among these cases [44, 45]. Despite this recommendation, the majority of patients could not be given adjuvant chemotherapy due to advanced age and associated comorbidities, which would limit the overall tolerance during chemotherapy [46]. Considering these features, methylene blue injection could play a major role in the appropriate lymph node detection, reducing uncertainty of staging resulting in under- or overtreatment.

### Limitations

Our study has limitations. One of our experienced pathologists refused to participate in the trial, and regardless of the methylene blue injection, he performed a fat-dissolving internal step to improve lymph node recognition. His cases had to be excluded from the study. Methylene blue dyeing may not be optimal for every pathologist, particularly if other methods of enhanced lymph node detection are used routinely already. Other techniques of lymph node harvesting (for example alcoholic fixation and fat-dissolving techniques) might have been used as controls. Additionally, the exact sizes of lymph nodes, especially positive lymph nodes, were not recorded in the pathological documentation. Therefore, we cannot comment on the efficacy of methylene blue injection on identification of smaller mesenteric lymph nodes. Furthermore, a detailed analysis of distinct clinical subgroups to assess effectivity of MB injection would have required an *a priori* power calculation for this purpose, and as such, statistically non-significant results in our subgroups may have stemmed from a type II statistical error.

## Conclusion

In our study, application of the methylene blue staining technique resulted in an increment of examined lymph node yield, and an improvement of lymph node staging with a 5-year overall survival benefit of patients with colorectal cancer undergoing colorectal resection. In conclusion, methylene blue staining technique seems a reasonable approach to be adapted for routine use in healthcare systems with limited staff and expenditure.

## Data Availability

The raw data supporting the conclusion of this article will be made available by the authors, without undue reservation.

## Abbreviations

CME, complete mesocolic excision; CRC, colorectal cancer; LN, lymph node; MB, methylene blue; NAT, neoadjuvant treatment; TME, total mesorectal excision; UICC, Union for International Cancer Control.
